# Optimal Classification of Atrial Fibrillation and Congestive Heart Failure Using Machine Learning

**DOI:** 10.3389/fphys.2021.761013

**Published:** 2022-02-03

**Authors:** Yunendah Nur Fuadah, Ki Moo Lim

**Affiliations:** ^1^Computationa Medicine Lab, Department of IT Convergence Engineering, Kumoh National Institute of Technology, Gumi, South Korea; ^2^Computational Medicine Lab, Department of Medical IT Convergence Engineering, Kumoh National Institute of Technology, Gumi, South Korea

**Keywords:** atrial fibrillation, congestive heart failure, Hjorth descriptor, entropy-based features, machine learning

## Abstract

Cardiovascular disorders, including atrial fibrillation (AF) and congestive heart failure (CHF), are the significant causes of mortality worldwide. The diagnosis of cardiovascular disorders is heavily reliant on ECG signals. Therefore, extracting significant features from ECG signals is the most challenging aspect of representing each condition of ECG signal. Earlier studies have claimed that the Hjorth descriptor is assigned as a simple feature extraction algorithm capable of class separation among AF, CHF, and normal sinus rhythm (NSR) conditions. However, due to noise interference, certain features do not represent the characteristics of the ECG signals. This study addressed this critical gap by applying the discrete wavelet transform (DWT) to decompose the ECG signals into sub-bands and extracting Hjorth descriptor features and entropy-based features in the DWT domain. Therefore, the calculation of Hjorth descriptor and entropy-based features performed on each sub-band will produce more detailed information of ECG signals. The optimization of various classifier algorithms, including k-nearest neighbor (k-NN), support vector machine (SVM), random forest (RF), artificial neural network (ANN), and radial basis function network (RBFN), was investigated to provide the best system performance. This study obtained an accuracy of 100% for the k-NN, SVM, RF, and ANN classifiers, respectively, and 97% for the RBFN classifier. The results demonstrated that the optimization of the classifier algorithm could improve the classification accuracy of AF, CHF, and NSR conditions, compared to earlier studies.

## Introduction

Atrial fibrillation (AF) is one of the most common sustained arrhythmias affecting 59.7 million people in 2019, more than two times the number of cases reported in 1990 (Roth et al., 2020). Meanwhile, congestive heart failure (CHF) is an increasingly frequent cardiovascular disease that affects 64.34 million cases according to the current worldwide prevalence in 2017 (Lippi and Sanchis-Gomar, 2020). As cardiovascular disorders affect millions of people and potentially lead to death, AF and CHF have become a major public health concern worldwide (Savarese and Lund, 2002). Early diagnosis of AF and CHF could potentially prevent the long-term complications and sudden cardiac death.

Researchers have used a non-invasive method by measuring and analyzing the ECG characteristics, which strongly correlate with cardiovascular conditions (Krittanawong et al., 2020; Taye et al., 2020). Therefore, in recent decades, there has been a significant increase in interest in the field of automatic classification of cardiovascular disorders (Sharma et al., 2019; Jeong et al., 2021), including AF and CHF, based on ECG signals and machine learning approaches (Acharya et al., 2008; Rizal and Hadiyoso, 2015; Hadiyoso and Rizal, 2017; Yingthawornsuk and Temsang, 2019; Faust et al., 2020; Krittanawong et al., 2020).

Ping and Chen (2020) used long short-term memory ECG signals and convolutional neural network (CNN) to classify AF and normal conditions and obtained an f1-score value of 89.55%. Similarly, Sidrah and Dashtipour (2020) used CNN to classify AF and normal conditions and obtained a classification accuracy of 86.5%. Meanwhile, Nurmaini and Tondas (2020) reported 99.17% accuracy using the CNN model to classify ECG signals into three conditions, namely, AF, non-AF, and normal conditions.

Sharma et al. (2018) reported 93.33% accuracy using heart rate variability (HRV) features and support vector machine (SVM) to classify CHF and normal sinus rhythm (NSR). Similarly, Li (2020) used HRV features with the multifractal fluctuation analysis to analyze the heartbeat signal of CHF conditions. Ning et al. (2021) extracted features from the R-R interval (the interval between R peaks) interval sequence, computed the time spectra of the ECG signal, and used a hybrid deep learning algorithm composed of CNN with recurrent neural network (RNN) to classify CHF and NSR. Their proposed method provided 99.93% accuracy. Moreover, Porumb et al. (2020) used raw ECG heartbeat as the input of the CNN model and obtained 100% accuracy. Their study revealed that the morphological characteristics of ECG signals are the most important information to identify CHF conditions.

The aforementioned studies developed a binary classification system to classify AF and NSR or CHF and NSR. Therefore, several studies classify ECG signals into three conditions, namely, AF, CHF, and NSR. Rizal and Hadiyoso (2015) and Hadiyoso and Rizal (2017) used the Hjorth descriptor approach to evaluate ECG signals based on activity, mobility, and complexity features. Several classifier algorithms used in the classification process included k-mean clustering, k-nearest neighbor (k-NN), and multilayer perceptron and obtained 88.67, 99.3, and 99.3% accuracy, respectively, in 2015 and 94% accuracy using the k-NN classifier in 2017 (Hadiyoso and Rizal, 2017).

Furthermore, Yingthawornsuk and Temsang (2019) used 90 recordings from the primary datasets of these three conditions and provided 84.89, 88.22, and 76% accuracy using least-squares (LS), maximum likelihood (ML), and SVM, respectively. Their results demonstrated that the Hjorth descriptor efficiently separated groups with cardiac arrhythmia.

Apart from using statistical approaches such as Hjorth descriptor for feature extraction to represent AF, CHF, and NSR conditions, several researchers used entropy-based features to extract information from ECG signals. Zhao et al. (2018) proposed an entropy-based AF detector, which obviously distinguishes AF and non-AF by providing the performance area under the curve (AUC) score of 0.981. Tang et al. (2017) used sample entropy as one of the features in detecting AF signals and obtained the AUC score of 0.972. Moreover, Hussain et al. (2020) reported an AUC score of 0.97 using entropy-based features with an SVM classifier to classify CHF conditions. In addition, Yoon et al. (2017) calculated entropy features from ECG signals such as Shannon entropy and sample entropy features and showed that the entropy features successfully represented AF, CHF, and NSR by providing the classification accuracy of 91.08%.

The most challenging aspect of classifying ECG signals is the feature extraction process. The aforementioned studies (Rizal and Hadiyoso, 2015; Hadiyoso and Rizal, 2017; Yingthawornsuk and Temsang, 2019) used a statistical approach by applying the Hjorth descriptor method to extract features from ECG signals. However, certain limitations of earlier studies that used Hjorth descriptors include susceptible noise that impacts the value of variance or activity in Hjorth descriptor features. Therefore, the statistical approach is insufficient to extract the features perfectly and represent the information contained in ECG signals.

Another limitation of the earlier studies is not implementing signal decomposition before calculating the features that potentially improve the performance accuracy (Rizal and Hadiyoso, 2015). Discrete wavelet transform (DWT) is an essential tool widely used to analyze the nonstationary signal. DWT is provided to extract the features in the time-frequency domain. In addition, DWT decomposes the signal into several sub-bands consisting of approximation (low frequency) and detail component (high frequency). Extracting the features separately in each sub-band will generate more detailed information on ECG signals. The study proposed by Chashmi and Amirani (2019) for arrhythmia classification reported an accuracy of 99.83% by applying DWT to decompose the ECG signal.

To overcome certain limitations of the aforementioned studies, we proposed a new approach by applying the DWT to decompose the ECG signal into sub-bands prior to applying Hjorth descriptor features and adding entropy-based features in the DWT domain as the feature extraction method. The statistical approach using Hjorth descriptor (activity, mobility, and complexity) and entropy-based features, including Shannon entropy, sample entropy, permutation entropy, dispersion entropy, bubble entropy, and slope entropy, potentially extracts the features of ECG signals perfectly and represents the condition of AF, CHF, and NSR. Furthermore, the optimization of various classifier methods that commonly used to classify ECG signals in the previous studies, including k-NN, SVM, random forest (RF), artificial neural network (ANN), and radial basis function network (RBFN), was investigated to improve the performance accuracy in classifying AF, CHF, and NSR conditions.

## Methods

**FIGURE 1:**
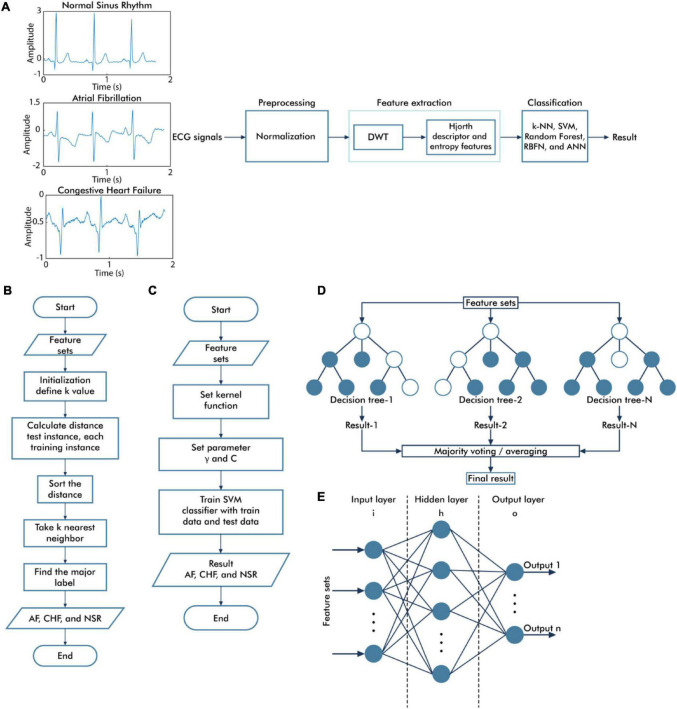
Overall diagram of the proposed system. The general block diagram of the classification system based on discrete wavelet transform and Hjorth descriptor feature extraction **(A)**, the flowchart of the *k*-nearest neighbor algorithm **(B)**, the flowchart of the support vector machine algorithm **(C)**, the topology of random forest classifier algorithm **(D)**, and the architecture of neural network algorithm **(E)**.

In this study, we applied DWT to the decomposition signal prior to calculating the Hjorth descriptor features and entropy-based features as the feature extraction method, followed by several classifier algorithms such as k-NN, SVM, RF, ANN, and RBFN (Figure 1A).

### Dataset

The ECG signal data comprised three conditions, namely, NSR from MIT-BIH Normal Sinus Rhythm Database (Moody, 1999), AF from MIT-BIH Atrial Fibrillation Database (Moody and Mark, 1983), and CHF from BIDMC Congestive Heart Failure Database (Baim et al., 1986). The dataset was created from the original data, with a sampling rate of 250 Hz, and each file comprised 2–3 cycles of QRS of the ECG signals. Each class consists of 50 files of ECG signals; therefore, there are 150 ECG signals divided into 112 train data and 38 test data.

### Preprocessing

In the preprocessing step, the ECG signal amplitude was normalized. If the ECG signal is *x* (*n*), where *n* = 1, 2, …, *N*, the value of *N* indicates the length of the signal; then, the signal is normalized using the following equations (Rizal and Hadiyoso, 2015; Hadiyoso and Rizal, 2017):


(1) $$y\,\left(n\right)=x\,\left(n\right)-\frac{1}{N}\,\sum_{i=1}^{N}x\,\left(n\right)$$

(2) $$z\,\left(n\right)=\frac{y\,\left(n\right)}{m\,a\,x\,\left|y\,\left(n\right)\right|}$$

The amplitude of the *z*(*n*) signal is -1 to +1; therefore, the difference in the amplitude range of the signal due to differences in signal recording can be eliminated. The normalization process did not change the morphology of the ECG signal, which is used in differentiating AF, CHF, and NSR conditions. In addition, normalization is an essential stage of data preparation in machine learning which can transform different data into the same scale to standardize the data as the input of feature extraction and classification process.

### Discrete Wavelet Transform

**FIGURE 2:**
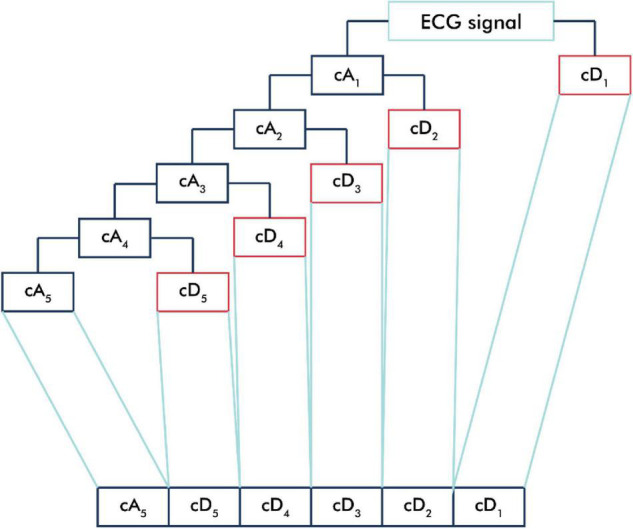
One-dimensional wavelet decomposition. The ECG signal is passed into the low-pass filter (LPF) to produce an approximation component (cA) and is passed into the high-pass filter (HPF) to produce a detailed component (cD). In one-dimensional wavelet decomposition, five-level decomposition generated six sub-bands, which comprised one cA and five cD sub-bands.

The ECG signal was passed into the low-pass filter (LPF) and high-pass filter (HPF) according to the mother wavelet used and then downsampled (Rizal, 2015). The output of the LPF produced an approximation component (cA), and the output of the HPF produced a detailed component (cD) (Kociolek et al., 2001). In this study, we applied a five-level decomposition that generated six sub-bands, which comprised one cA and five cD sub-bands (Figure 2).

We calculated the Hjorth descriptor parameters such as activity, mobility, and complexity for each sub-band. Furthermore, we also calculated entropy-based features such as Shannon entropy, sample entropy, permutation entropy, dispersion entropy, bubble entropy, and slope entropy for each sub-band. Therefore, 54 features were generated as input for the classifier algorithms.

### Hjorth Descriptor

Consider *x*(*n*)as a signal, for *n* = 0,1,2,3,…,*N*−1. Then, *x*(*n*)′ can be defined as the first difference of the signal as shown in the following equation (Mouzé-Amady and Horwat, 1996; Rizal et al., 2015; Grover and Turk, 2020):


(3) $$x\,{\left(n\right)}^{\prime }=x\,\left(n\right)-x\,\left(n-1\right)\,f\,o\,r\,n=0,1,2,3,\ldots ,N-1$$

Furthermore, *x*(*n*)″ is defined as the second difference of the signal as shown in the following equation:


(4) $$x\,{\left(n\right)}^{\prime \prime }=x\,{\left(n\right)}^{\prime }-x\,{\left(n-1\right)}^{\prime }\,f\,o\,r\,n=0,1,2,3,\ldots ,N-1$$

Consider σ_*x*_ as the SD of *x*(*n*). Then,σ_*x*1_ and σ_*x*2_ can be defined as the SD of *x*(*n*)′and *x*(*n*)″, respectively. The SD of *x*(*n*) can be calculated using the following equation:


(5) $$\sigma_{x}=\sqrt{\frac{\sum_{n=0}^{N-1}{\left(x\,\left(n\right)-\bar{x}\right)}^{2}}{N}}$$

(6) $$\text{where}\,\bar{x}=\frac{1}{N}\,\sum_{n=0}^{N-1}x\,\left(n\right).$$

Activity refers to the signal variation or the squared SD of the amplitude, as shown in the following equation (Mouzé-Amady and Horwat, 1996; Rizal et al., 2015; Grover and Turk, 2020):


(7) activity=variance=σx2

Mobility calculates the SD of the slope in relation to the SD of the amplitude, as shown in the following equation:


(8) $$\text{mobility}=M_{x}=\frac{\sigma_{x\,1}}{\sigma_{x}}$$

Complexity measures the number of standard slopes generated in the average time to generate one standard amplitude, as determined by the mobility shown in the following equation:


(9) complexity=F⁢F=Mx′Mx=σx⁢2σx⁢1σx⁢1σx

### Entropy

Entropy is a method for calculating the uncertainty of information contained in the systems. Shannon entropy is a measure of uncertainty associated with random variables based on the probability distribution of energy which can be calculated using the following equation (Li and Zhou, 2016):


(10) $$S\,h\,a\,n\,E\,n\,\left(X\right)=-\sum_{i}p_{i}.\ln\left(p_{i}\right)$$

where *X* = *xi*, *i* = 1,…, *N* is a time series and *p_i_* represents the time-series probability.

Sample entropy is a negative natural logarithmic of the sequence of probability data vector in time series as shown in the following equation (Horie et al., 2018):


(11) $$\text{SampEn}=-l\,n\,\left[\sum_{i=1}^{N-m\,\tau }C_{m+1,i\,\left(r\right)}/\sum_{i=1}^{N-m\,\tau }C_{m,i\,\left(r\right)}\right]$$

where *C*_*m,i(r)*_ is the correlation integral representing the number of points in a distance *r* (filter threshold) from the *i*th point while embedding the signal in *m*-dimensional space, and τ represents the time lag.

Permutation entropy is an entropic measure according to the comparison between the neighboring values of a time series. The permutation entropy measures the diversity of the ordinal pattern distribution which could be defined by the following equation (Xia et al., 2018):


(12) $$\text{PermEn}=-\sum_{i}p\,\left(\pi \right)\,\ln p\,\left(\pi \right)$$

where π represents an ordinal pattern, and *p*(π) represents the relative frequency of each π.

Dispersion entropy is a powerful and fast approach to measure the randomness of the signal, which can explore the amplitude and frequency changes of the signal simultaneously. The dispersion entropy can be calculated based on Shannon entropy represented in the following equation, with *m* as embedding dimension and *a* as the number of classes (Shi et al., 2020):


(13) $$\text{DispEn}=-\sum_{i=1}^{a^{m}}p\,\left(\pi_{u_{0}\,u_{1}\,\ldots \,u_{m-1}}\right)\,\ln\,\left(p\,\left(\pi_{u_{0}\,u_{1}\,\ldots \,u_{m-1}}\right)\right)$$

Bubble entropy is an entropic measure based on permutation entropy (Manis et al., 2017). The bubble entropy can be calculated using the following equation as the normalized difference of the entropy of the swaps needed to sort the vectors of lengths *m* +1 and *m*:


(14) $$b\,E\,n=\left(H_{s\,w\,a\,p\,s}^{m+1}-H_{s\,w\,a\,p\,s}^{m}\right)/\log\left(\frac{m+1}{m-1}\right)$$

(15) Hs⁢w⁢a⁢p⁢sm=-log⁢∑i=0(m2)pi2

Meanwhile, slope entropy is a new statistical measurement that includes amplitude information in symbolic representation from the time-series input (Cuesta-Frau, 2019).

### Classifier Algorithms

The general process of the k-NN algorithm is illustrated in Figure 1B. The classification performance of the k-NN algorithm depends on the features used as the input of the k-NN algorithm and the *k*-value of the k-NN algorithm. The optimization was performed using the grid search method to select the best parameter, including the best *k*-value selection for varying values of *k* (1–31), and the distance matrices including Euclidean, Minkowski, and Chebyshev.

Figure 1C illustrates the general process of the SVM algorithm. Three types of kernel functions, namely, linear, radial basis function (RBF), and polynomials, were applied in this study. The optimization procedure of the SVM algorithm was evaluated using the grid search method to find the best γ parameter selection of the linear, RBF, and polynomial kernel functions (from 1e–01 to 1e–06) and the best regularization parameter *C* selection for SVM (from 1 to 1e + 05).

The topology of the RF classifier is represented in Figure 1D. RF is known as an ensemble of decision tree classifier (Pandey et al., 2020). In the classification process, all of the trees give a class vote, and RF will classify the input based on the majority vote. The optimal parameter of RF is selected using the grid search method to determine the best number of trees and the best criterion that provides the best performance result.

An ANN is a fully connected structure that includes three main layers, namely, input, hidden, and output layers (Figure 1E). The feature extraction results were assigned as inputs for the ANN architecture. Therefore, there are 54 nodes in the input layer. The hidden layer processes the input of the previous layers and transfers the result to the output nodes. In determining the number of nodes in the hidden layer, there are several theories regarding this case, which are the number of nodes must be between the input layer size (54 nodes) and the output layer size (3 nodes) (Blum, 1992), the number of nodes in the hidden layer is around 70–90% of the input size (Boger and Guterman, 1997), and the number of nodes should be no greater than two times as much as the input layer (Swingler, 1996). In this study, we proposed the simple ANN model, which consists of one hidden layer, with the number of nodes in the hidden layer equal to 32. The rectified linear unit (ReLU) activation function was applied for the hidden layer, and the softmax activation function was applied to the output layers, which comprised three nodes representing the AF, CHF, and NSR conditions. While training the model, we used categorical cross-entropy as a loss function and Adam as optimizer algorithms to minimize error during training with a learning rate of 0.001 and 200 epochs.

The topology of RBFN is generally similar to the structure of feed-forward ANNs, as shown in Figure 1E. However, there are fundamental differences, such as the RBFN has only one hidden layer and the activation function of the hidden layer uses a radial basis activation function (Satapathy et al., 2019). While training the model, we used 32 nodes in the RBFN layer, mean square error as a loss function, RMSprop as an optimizer algorithm with a learning rate of 0.001 and 500 epochs.

## Results

**FIGURE 3:**
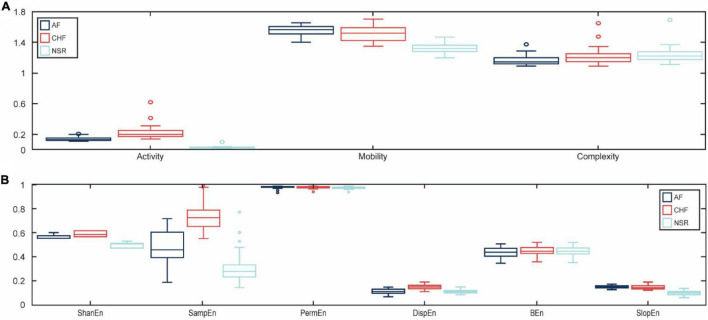
The boxplot distributions for each feature. The distribution value of Hjorth descriptor features for atrial fibrillation (AF), congestive heart failure (CHF), and normal sinus rhythm (NSR) conditions **(A)** and the distribution value of entropy-based features for AF, CHF, and NSR conditions **(B)**.

In this study, we used the ANOVA test for the statistical analysis of the difference between features for AF, CHF, and NSR conditions with statistical significance at p<0.05. As explained in Table 1, Hjorth descriptor features obtained statistical significance with *p*-value (p<0.05). Moreover, the boxplot distributions of the conditions of Hjorth descriptor features are shown in Figure 3A. Based on Figure 3A, activity and mobility features obviously can differentiate between AF, CHF, and NSR conditions. Meanwhile, the complexity feature showed a slight overlapping between AF, CHF, and NSR conditions. However, the *p*-value of the complexity feature was 5.81e–05 (p<0.05), which means the feature was statistically significant to differentiate between AF, CHF, and NSR conditions.

**TABLE 1:** Mean and *p*-values of features for atrial fibrillation (AF), congestive heart failure (CHF), and normal sinus rhythm (NSR) conditions.

Feature extraction method	Features	AF Mean ± std	AF Mean ± std	NSR Mean ± std	*p*-value
Hjorth descriptor	Activity	0.127 ± 0.033	0.230 ± 0.101	0.026 ± 0.015	4.54e–34
	Mobility	1.557 ± 0.061	1.511 ± 0.097	1.326 ± 0.063	1.37e–33
	Complexity	1.151 ± 0.060	1.204 ± 0.095	1.225 ± 0.090	5.81e–05
Entropy-based features	Shannon entropy	0.656 ± 0.020	0.591 ± 0.020	0.506 ± 0.033	1.5e–63
	Sample entropy	0.509 ± 0.126	0.768 ± 0.108	0.323 ± 0.115	1.14e–40
	Permutation entropy	0.991 ± 0.006	0.990 ± 0.007	0.984 ± 0.018	2.27e–03
	Dispersion entropy	0.135 ± 0.012	0.156 ± 0.011	0.133 ± 0.009	5.77e–23
	Bubble entropy	0.429 ± 0.035	0.437 ± 0.031	0.437 ± 0.032	3.83e–01
	Slope entropy	0.140 ± 0.006	0.139 ± 0.011	0.109 ± 0.010	3.61e–40

In addition, the *p*-value of entropy-based features was statistically significant by obtaining p<0.05 as shown in Table 1. However, bubble entropy obtained a *p*-value of 0.382 (p>0.05) which means the feature is statistically insignificant to differentiate AF, CHF, and NSR conditions. As shown in Figure 3B, the boxplot distribution of entropy-based features showed that mostly the entropy-based features can distinguish AF, CHF, and NSR conditions. Meanwhile, permutation entropy and bubble entropy showed similar values for each condition.

We applied fivefold cross-validations to get the best model in training the model. Furthermore, the optimization of each classifier algorithm is performed using the grid search method to find the best parameter that provides the highest classification accuracy. A total of 38 test data that included 12 AF, 14 CHF, and 12 NSR data were used to evaluate the system performance. Table 2 summarizes the classification accuracy of each feature set for each algorithm.

**TABLE 2:** Classification performance of each feature set.

Feature sets	Classification accuracy (%)				
	k-NN	SVM	Random forest	ANN	RBFN
Hjorth descriptor features	92	92	95	95	97
Entropy features	100	89	97	100	97
Hjorth descriptor and entropy features	100	100	100	100	97

For the k-NN classifier algorithm, optimization was conducted by selecting the best *k*-values (*k* = 1, 3, 5, 7, …, 31) and the distance matrices (i.e., Euclidean, Chebyshev, and Minkowski) using the grid search method. The optimal value of *k* = 1 obtained with the Minkowski distance was selected as the best parameter of the k-NN algorithm that provided the highest accuracy. The performance accuracy of the k-NN method reached 92% using the Hjorth descriptor features (i.e., activity, mobility, and complexity) and obtained an accuracy of 100% using entropy-based features (Shannon entropy, sample entropy, permutation entropy, dispersion entropy, bubble entropy, and slope entropy) as well as using a combination of Hjorth descriptor and entropy-based features which provided an accuracy of 100%.

For the SVM classifier algorithm, optimization was performed using the grid search method to find the best kernel (RBF, linear, and polynomial), the best γ parameter (from 1e–01 to 1e–06), and the best regularization parameter *C* (from 1 to 1e + 05). The best values of *C* = 1,000 and γ = 0.01 with an RBF kernel were selected as the optimal parameters to provide the highest accuracy. The performance accuracy of SVM with RBF kernel obtained 92% using Hjorth descriptor features, provided an accuracy of 89% using entropy-based features, and provided an accuracy of 100% using a combination of Hjorth descriptor and entropy-based features. In addition, the performance accuracy of SVM with linear kernel obtained 95% using Hjorth descriptor features, provided an accuracy of 92% using entropy-based features, and provided an accuracy of 95% using a combination of Hjorth descriptor and entropy-based features. Meanwhile, the performance accuracy of SVM with polynomial kernel obtained an accuracy of 63% using Hjorth descriptor features, obtained an accuracy of 89% using entropy-based features, and obtained an accuracy of 97% using a combination of Hjorth descriptor and entropy-based features.

The RF classifier algorithm optimization was conducted using the grid search method to find the best number of trees (from 10 to 500) and the best criterion (Gini and entropy) which provide the highest classification accuracy. The number of trees equals 150, and the criterion “Gini” was selected as the best parameter of RF that provides the highest classification accuracy. The classification accuracy of RF obtained 95% using Hjorth descriptor features, obtained an accuracy of 97% using entropy-based features, and obtained an accuracy of 100% using a combination of Hjorth descriptor and entropy-based features. The ANN classifier algorithm provided an accuracy of 95% using Hjorth descriptor features, provided an accuracy of 100% using entropy-based features, and used all combination features. Meanwhile, the RBFN classifier algorithm provided an accuracy of 97% using Hjorth descriptor features, entropy-based features, and combination of all features, respectively.

According to the results, the combination of Hjorth descriptor features and entropy-based features successfully extracted the information contained in the ECG signal. Theoretically, the ECG signal is a complex signal which has the complexity characteristic. There are several approaches to measure the complexity signals. The statistical approach, such as the Hjorth descriptor, which measures activity, mobility, and complexity, is commonly used to analyze the complexity of the signal. However, the statistical approach is insufficient to extract all of the information in ECG signals. Therefore, we need entropy-based features to extract more detailed information associated with the characteristics of AF, CHF, and NSR conditions. The classification performance improved significantly using Hjorth descriptor features and entropy-based features.

The performance accuracy after applying DWT, Hjorth descriptor features, and entropy-based features using the k-NN, SVM, RF, and ANN classifier algorithm achieved the accuracies of 100%, respectively, and achieved an accuracy of 97% using the RBFN classifier algorithm. This result outperformed the system performance achieved in earlier studies, which also used the Hjorth descriptor as a feature extraction method to classify AF, CHF, and NSR which achieved the accuracy of 99.3% (Rizal and Hadiyoso, 2015) and 94% using k-NN (Hadiyoso and Rizal, 2017) and an accuracy of 76% using SVM (Yingthawornsuk and Temsang, 2019). The result also outperformed the previous study, which used entropy-based features to classify AF, CHF, and NSR that obtained an accuracy of 91.08% (Yoon et al., 2017).

Furthermore, the accuracy performance of the proposed method was compared with several related works that also developed a system to detect the occurrence of AF and CHF in ECG signals. The previous studies showed good performance accuracy in classifying AF and normal conditions using the CNN model that obtained the f1-scores of 89.55% (Ping and Chen, 2020) and an accuracy of 86.5% (Sidrah and Dashtipour, 2020). Meanwhile, the highest performance accuracy in classifying CHF and normal conditions was performed using various methods, such as HRV features and SVM classifier, which obtained an accuracy of 93.33% (Sharma et al., 2018), an accuracy of 99.93% using CNN composed with RNN model (Ning et al., 2021), and an accuracy of 100% using CNN model (Porumb et al., 2020). However, the aforementioned studies are the binary classification that classifies AF and normal conditions or classifies CHF and normal conditions. Therefore, the results of this study, which can classify the condition of ECG signals into three conditions (AF, CHF, and NSR), can be concluded to provide a promising contribution for further development in ECG classification.

## Discussion

The broader objective of this study was to develop machine learning algorithms that would improve the accuracy performance achieved by the earlier studies in classifying AF, CHF, and NSR conditions (Rizal and Hadiyoso, 2015; Hadiyoso and Rizal, 2017). To achieve this objective, we applied the DWT, Hjorth descriptors, and entropy-based features as the feature extraction methods to generate the feature sets and trained them using several classifier algorithms, including SVM, k-NN, RF, ANN, and RBFN to recognize a set of features that are associated with a particular condition of ECG signal.

The feature extraction method based on statistical approach using Hjorth descriptor method obtained the promising classification accuracy performance using several classifier algorithms as shown in Table 2. However, the result using Hjorth descriptor features was still affected by false detection that influenced the performance of classification accuracy since the statistical approach could not perfectly extract the information of the ECG signal. In contrast, combining Hjorth descriptor features with entropy-based features significantly improved the classification accuracy up to 100% using the k-NN, SVM, RF, and ANN classifier algorithm and obtained an accuracy of 97% using the RBFN classifier algorithm. Hjorth descriptor features and entropy-based features successfully extracted the information of ECG signals and were appropriate to distinguish the conditions of ECG signals. Therefore, instead of using the Hjorth descriptor only as a feature extraction method, combining the Hjorth descriptor features with entropy-based features will provide significant information associated with the condition of ECG signals.

Based on the results, we can consider that the advantage of this study is successfully provided the highest classification accuracy performance in classifying AF, CHF, and NSR conditions of ECG signal. The improvement of the classification accuracy performance from the previous studies could be attributed to the application of the DWT method, the feature extraction method-based statistical approach using Hjorth descriptor and entropy-based features, and the extensive optimization for each classifier algorithm, including k-NN, SVM, RF, ANN, and RBFN classifier algorithm. However, the proposed model needs to be validated with a real and larger dataset for clinical implementation. We believe that it will improve even further when we use a more extensive dataset.

## Data Availability Statement

Publicly available datasets were analyzed in this study. This data can be found here: https://www.physionet.org/content/nsrdb/1.0.0/; https://www.physionet.org/content/afdb/1.0.0/; and https://www.physionet.org/content/chfdb/1.0.0/.

## Author Contributions

This study is the intellectual product of the entire team. Both authors contributed to programming the simulation source code, performing the simulation, and writing of the manuscript.

## Conflict of Interest

The authors declare that the research was conducted in the absence of any commercial or financial relationships that could be construed as a potential conflict of interest.

## Publisher’s Note

All claims expressed in this article are solely those of the authors and do not necessarily represent those of their affiliated organizations, or those of the publisher, the editors and the reviewers. Any product that may be evaluated in this article, or claim that may be made by its manufacturer, is not guaranteed or endorsed by the publisher.
